# Aerobic exercise prevents cardiomyocyte damage caused by oxidative stress in early cardiovascular disease by increasing vascularity while L-arginine supplementation prevents it by increasing activation of the enzyme nitric oxide synthase

**DOI:** 10.1590/1414-431X2023e12533

**Published:** 2023-08-14

**Authors:** J.M.C.R.J. Bertoldi, R. Kindlovits, H.N.M. Rocha, C. Fernandes-Santos, J.L.P. Gomes, E.M. Oliveira, I.C. Muniz, M.M. Santos, J.F. Pereira, N.G. Rocha, A.C.L. da Nóbrega, R.F. Medeiros

**Affiliations:** 1 Departamento de Fisiologia e Farmacologia, Universidade Federal Fluminense, Niterói, Rio de Janeiro, RJ, Brasil; 2 Instituto Nacional de Ciência e Tecnologia - (In)atividade física e exercício, Rio de Janeiro, RJ, Brasil; 3 Departamento de Ciências Básicas, Universidade Federal Fluminense, Nova Friburgo, Rio de Janeiro, RJ, Brasil; 4 Departamento de Biodinâmica do Movimento do Corpo Humano, Universidade de São Paulo, São Paulo, SP, Brasil; 5 Departamento de Nutrição e Dietética, Universidade Federal Fluminense, Niterói, Rio de Janeiro, RJ, Brasil

**Keywords:** Cardiovascular disease, Exercise, Arginine, Fructose, Oxidative stress

## Abstract

L-Arginine and chronic exercise reduce oxidative stress. However, it is unclear how they affect cardiomyocytes during cardiovascular disease (CVD) development. The aim of this research was to investigate the possible effects of L-arginine supplementation and aerobic training on systemic oxidative stress and their consequences on cardiomyocytes during cardiometabolic disease onset caused by excess fructose. Wistar rats were allocated into four groups: control (C), fructose (F, 10% fructose in water), fructose training (FT; moderate running, 50-70% of the maximal velocity), and fructose arginine (FA; 880 mg/kg/day). Fructose was given for two weeks and fructose plus treatments for the subsequent eight weeks. Body composition, blood glucose, insulin, lipid profile, lipid peroxidation, nitrite, metalloproteinase-2 (MMP-2) activity, left ventricle histological changes, microRNA-126, -195, and -146, eNOS, p-eNOS, and TNF-α expressions were analyzed. Higher abdominal fat mass, triacylglycerol level, and insulin level were observed in the F group, and both treatments reversed these alterations. Myocardial vascularization was impaired in fructose-fed groups, except in FT. Cardiomyocyte hypertrophy was observed in all fructose-fed groups. TNF-α levels were higher in fructose-fed groups than in the C group, and p-eNOS levels were higher in the FA than in the C and F groups. Lipid peroxidation was higher in the F group than in the FT and C groups. During CVD onset, moderate aerobic exercise reduced lipid peroxidation, and both training and L-arginine prevented metabolic changes caused by excessive fructose. Myocardial vascularization was impaired by fructose, and cardiomyocyte hypertrophy appeared to be influenced by pro-inflammatory and oxidative environments.

## Introduction

According to the World Health Organization, approximately 17.9 million people died from cardiovascular disease (CVD) worldwide in 2019, accounting for 32% of global mortality (1). Therefore, it is important to understand the causes and mechanisms of cardiovascular and metabolic impairments, as well as non-pharmacological methods to prevent the impact of CVD and metabolic disease.

Cardiovascular and metabolic damage is mainly related to modifiable risk factors, such as poor diet and sedentary habits (1). In this context, the consumption of industrialized products rich in fructose has been excessively high, as has the incidence of chronic non-communicable diseases (1,2). In recent years, chronic consumption of fructose has been associated with cardiometabolic damage, such as obesity and dyslipidemia (3), and also with increases in the levels of pro-inflammatory cytokines, such as tumor necrosis factor-alpha (TNF-α) and interleukin (IL)-6, and the generation of reactive oxygen species (ROS), which contribute to diabetic cardiomyopathy (4).

Evidence from literature indicates that increased levels of inflammatory mediators and ROS induce activation of the myocardial hypertrophy signaling pathway (5), cardiac remodeling by matrix metalloproteinase (MMP) activation, progressive cardiomyocyte loss by apoptosis, and decrease in genome stability (6). These damages could be mediated by changes in microRNA expression such as mRNA-126, -195, and -146a, which can lead to heart failure. Therefore, it is extremely important to know the main triggers of these phenomena and how to mitigate them (7).

Among low-cost and no-side-effect strategies to prevent the progression of cardiovascular disease, studies show that physical training and L-arginine supplementation promote several health benefits. It is already known that exercise training improves endothelial function by increasing vasodilator substance (i.e., nitric oxide [NO]) production (8) and insulin sensitivity (9). In addition, it has potent anti-inflammatory and antioxidant effects (10). It is also known that the amino acid L-arginine participates as a substrate in a reaction catalyzed by nitric oxide synthase (NOS) and is converted into nitric oxide (NO) (11). In addition, L-arginine is associated with inhibition of platelet aggregation, reduced leukocyte adhesion, improved inflammatory responses (12), and increased insulin sensitivity (13).

Hence, the hypothesis of this study was that both aerobic training and L-arginine supplementation are capable of reversing the pro-oxidative environment caused by excess fructose, preventing metabolic and molecular damage in cardiomyocytes. Therefore, the aim of this study was to investigate the systemic pro-oxidative environment caused by a fructose-rich diet, how it impacts cardiomyocytes, and whether aerobic training and L-arginine supplementation prevent molecular damage in cardiomyocytes, thereby avoiding CVD progression.

## Material and Methods

The study protocol was approved by the Ethics Committee for the Care and Use of Laboratory Animals of Fluminense Federal University (Protocol 871/16). Male Wistar rats (329.9±8.9 g, 2 months old, 7 per group) were housed in cages (3-4 rats per cage) with a controlled room temperature (25±1°C) and 12-h light/dark cycle (lights on at 7 AM and off at 7 PM). All procedures were performed in the morning.

Twenty-eight animals were randomly allocated into two groups: control (C) group, which received commercial chow (Nuvlab Cr-1, Nuvital Nutrients, Brazil) and water *ad libitum* for 10 weeks, and fructose (F) group, which received commercial chow and 10% fructose (Sigma Aldrich, USA) diluted in water *ad libitum* for 10 weeks. After the first two weeks of fructose feeding, the animals showed increased insulinemia, body fat, serum triglycerides, and oxidative stress, as previously described by this group (14). Then, animals from the F group were subdivided into three experimental groups: F group; fructose arginine group (FA), which received L-arginine supplementation (880 mg/kg per day via orogastric gavage); and fructose training group (FT) (50-75% maximal running speed on treadmill, 4 days/week).

During the entire experiment, water and food intake was monitored twice a week. Caloric intake calculation took into consideration the amount of carbohydrates and proteins in grams multiplied by 4 kcal and that of lipids multiplied by 9 kcal. Fructose ingestion was considered to be 4 kcal/g.

### L-Arginine supplementation

Animals in the FA group received L-arginine supplementation (Sigma Aldrich) at 880 mg/kg per day via orogastric gavage for 8 weeks. The L-arginine dose was calculated according to a formula based on body surface area (15), assuming that the dose was equivalent to 10 g of the amino acid for an adult human (16). All animals that did not receive L-arginine gavage received vehicle gavage (dimethyl sulfoxide - DMSO) in the same proportion.

### Aerobic training

All animals underwent an adaptation period for 4 weeks with low-intensity treadmill exercise (Inbrasport, Brazil) (0.3 km/h) 5 min per day, once a week.

The procedure was maintained throughout the experiment for the groups that did not undergo physical training. To determine the initial maximum aerobic capacity and prescribe physical training, all animals individually underwent a maximal exercise test (MET) on a treadmill with 11% inclination, an initial velocity of 1 km/h, and velocity increments of 0.1 km every 2 min until exhaustion.

Animals in the FT group underwent aerobic training. The protocol consisted of 1 h of moderate aerobic exercise per day, 4 days a week, with alternating non-training days throughout the weeks of the protocol, on a treadmill with 0% inclination and 50-75% of the maximum speed reached in MET (17).

### Euthanasia

Seventy-two hours after the final exercise session, all animals were anesthetized intraperitoneally with ketamine (40 mg/kg) and xylazine (8 mg/kg) (Virbac Laboratory S.A., Brazil) and euthanized by cardiac puncture (18). Blood collection was performed during the final experimental week, after 5 h of fasting. Blood was collected and immediately centrifuged at 3500 *g* for 15 min at room temperature, and the serum was stored at -80°C. KCL was infused into the heart to ensure that the hearts of all animals were collected at the time of diastole. The heart was then removed, and the left ventricle (LV) dissected, abdominal fat and lean mass were collected in this order, weighed, and the tissues were stored at -80°C for posterior analysis (19).

### Total cholesterol, high-density lipoprotein cholesterol (HDL-c), and triacylglycerol

These analyzes were performed on serum using Labtest enzymatic colorimetric kits (Brazil) as previously described (20).

### Low-density lipoprotein cholesterol (LDL-c)

The quantification of LDL-c was performed in serum and estimated indirectly using the Friedewald formula (21), as described.

### Glucose analysis

Blood glucose was measured in blood collected from the tail, before which the animals received only tap water *ad libitum,* using an automatic glucometer (Accu-Check Advantage, Roche Diagnostics, Switzerland).

### Insulin analysis

Serum insulin levels were measured in blood collected at the end of the protocol, using a 96-well enzyme-linked immunosorbent assay kit (cat. #EZRMI-13K, EMD Millipore Corporation, Germany).

### Nitrite

Serum previously stored at -80°C was used for indirect analysis of NO. To measure serum nitrite concentrations, samples were injected into an acidified triiodide solution. This reduced the nitrite present in the sample to NO, which was detected using a NO analyzer by chemiluminescence (Sievers Model 280 NO Analyzer, GE Healthcare, USA) (22).

### Lipid peroxidation

Malondialdehyde production was quantified in serum and the reaction was based on the measurement of the pink chromogen formed by the reaction of malondialdehyde with two molecules of thiobarbituric acid, in an acidic environment and at high temperature, a reaction called “thiobarbituric acid reactive substances” (TBARS). Quantification of malondialdehyde was performed by spectrophotometry with absorbance at 532 nm (23).

### Histological analysis

LV samples were fixed in formaldehyde, sectioned (3-μm thick), and stained with hematoxylin and eosin. Digital images were acquired using an Evos XL Core system (Thermo Fisher Scientific, USA). Stereological analysis was performed by a blinded researcher using STEPanizer (24). Cardiomyocyte cross-sectional area and the volume density of cardiomyocytes and capillaries were determined to estimate the vessel/cardiomyocyte volume ratio to estimate tissue perfusion.

### MMP-2 activity

**Figure 1:**
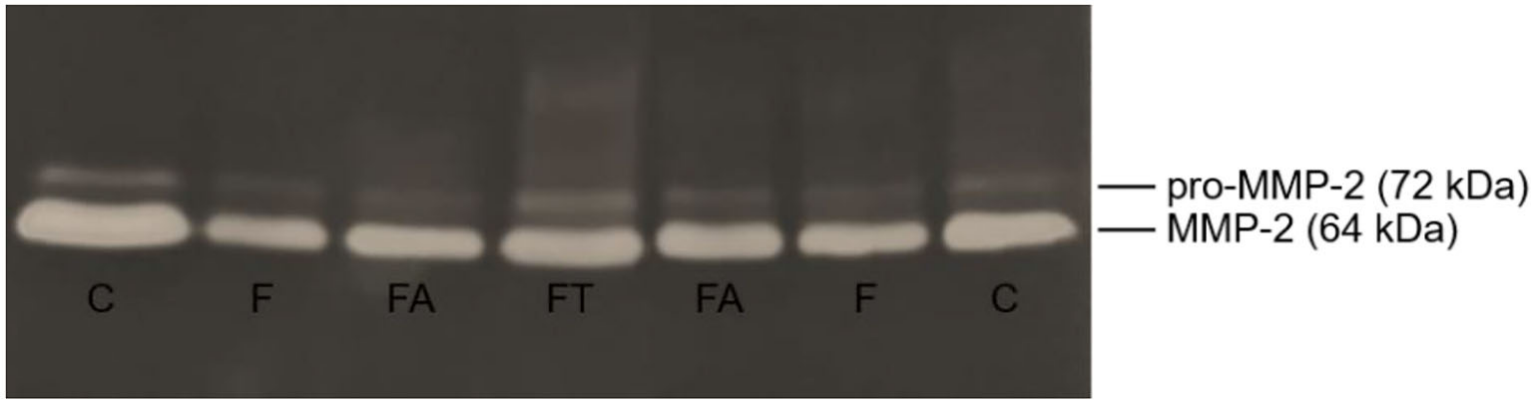
Representative image of zymography gel. Groups: C: control; F: fructose; FT: fructose training; FA: fructose arginine. pro-MMP-2: inactive metalloproteinase-2 (72 kDa); MMP-2: active metalloproteinase-2 (64 kDa).

Serum MMP-2 activity was determined by zymography according to Storch et al. (25). Briefly, serum samples were subjected to electrophoresis in a 12% polyacrylamide gel and copolymerized with 1% gelatin. An internal standard (control serum sample) and a protein molecular weight marker (Bio-Rad, USA) were used to analyze and compare the gels. Gels were stained with 5% Coomassie blue R-250 Brilliant Blue (Sigma-Aldrich) and destained with a solution of 50% methyl alcohol and 10% glacial acetic acid. Scion Image software (Scion Corporation, USA) was used by a blinded researcher to quantify band intensity; the more intense the band, the higher the enzyme activity. Inactive (pro-MMP-2) and active forms of MMP-2 were identified as bands at 72 and 64 kDa, respectively. Figure 1 shows a representative image of the zymography gel.

### MicroRNA quantification

MicroRNA-126, -195, and -146a were quantified in the LV. For total RNA purification, 100 mg of LV was homogenized in 1 mL of Tryzol (Invitrogen, USA), followed by extraction according to the manufacturer's instructions. Total RNA concentration was verified using a NanoDrop spectrophotometer (NanoDrop Technologies, USA).

RNA integrity was verified by immersion in TAE buffer and an electrophoretic run at 110 V on 1% agarose gel with 0.5 μg/mL ethidium bromide; images were obtained using ChemiDoc MP (Bio-Rad) equipment. Sample viability was verified by the integrity of the bands corresponding to the ribosomal RNA 18S and 28S subunits.

Complementary DNA (cDNA) was obtained from total RNA using specific primers for each microRNA, according to the TaqMan microRNA Assay protocol (Applied Biosystems, USA).

MicroRNA quantification was performed by real-time polymerase chain reaction (RT-qPCR) with TaqMan microRNA Assay 20X (Applied Biosystems) using specific microRNA assays: microRNA-126 (assay ID 002228), microRNA-195 (000494), and microRNA-146a (462788_mat). The PCR mix was incubated for 10 min at 95°C, followed by 40 cycles of 15 s at 95°C and 1 min at 60°C. Fluorescence was read on an ABI PRISM 7500 Detector (Applied Biosystems). The U6 gene (assay ID 001973) was used as an internal control (26).

### Western blotting

LV samples (100 mg) were homogenized in pH 6.4 lysis buffer (50 mM HEPES, 1 mM MgCl_2_, 10 nM EDTA, 1% Triton X) containing a protease and phosphatase inhibitor cocktail (Roche Applied Science, Germany). The protein concentration in each sample was quantified using the Bradford protein dosage method (27).

Thirty micrograms of LV protein extract was used to measure eNOS, eNOS-p, and TNF-α expression. Samples were pipetted in a 10% polyacrylamide gel and subjected to electrophoresis from 90 to 110 V. Afterward, the samples were transferred to a polyvinylidene fluoride membrane in a humid system (Bio-Rad Laboratories) at 250 mA for 120 min.

Membrane-adhered proteins were incubated with a solution containing 3% bovine serum albumin (INLAB, Brazil) in pH 7.6 Tris-saline buffer for 1 h at room temperature to block nonspecific sites on the membrane. Membranes were then incubated overnight at 4°C with primary antibodies (Santa Cruz Biotechnology, USA): anti-NOS3 (1:500, cat. #sc-376751), anti-eNOS-p (phosphorylation at Serine 1177) (1:250, sc-12972), and anti-TNF-α (1:500, sc-1350); or internal controls: anti-cyclophilin (1:1000, sc-20361) or anti-β-actin (1:500, sc-47778).

Subsequently, membranes were washed, incubated, and visualized using the Lumigen ECL PS-3 detection reagent (Amersham Biosciences Inc., USA), following the manufacturer's protocol. Images obtained by ChemiDoc MP (Bio-Rad) were quantified by densitometry using ImageJ software (version 1.44, NIH, USA).

### Statistical analysis

The appropriate sample size of seven animals per group was calculated using serum insulin as the primary outcome, assuming an average difference of 5% (effect size) and an equal SD of 5%. Both values were obtained from preliminary experiments by setting the power of the statistical test to 0.8 and the α error to 0.05.

The normality of the data was verified using the Shapiro-Wilk test. Differences among groups were tested by one-way ANOVA followed by Student's-Newman-Keuls *post hoc* test when appropriate. Data are reported as means±SE. Statistical significance was set at P<0.05. Statistical analyses were performed using GraphPad Prism software (version 5.0; GraphPad Software, Inc., USA).

## Results

### Sample characterization

**Figure 2:**
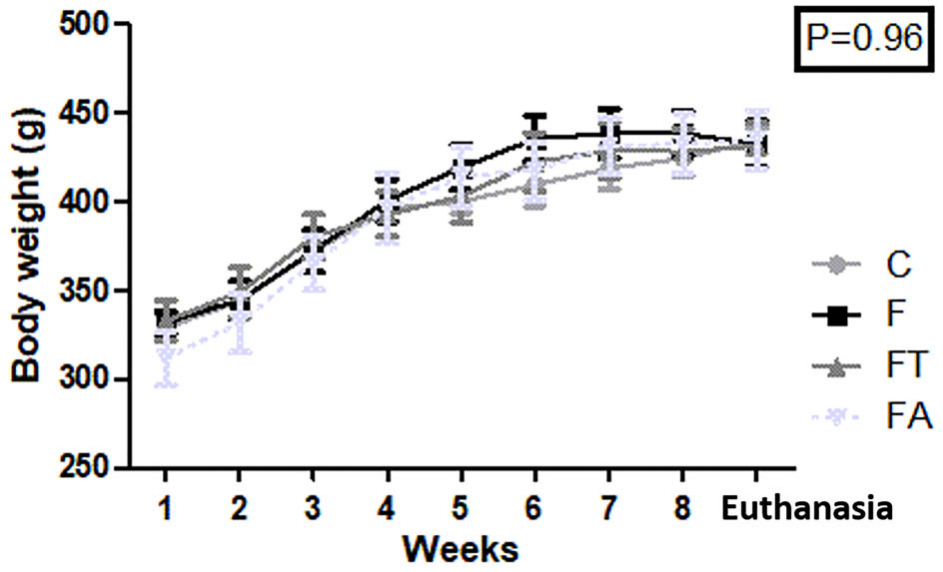
Body weight evolution through the experimental period (n=7 per group). Groups: C: control; F: fructose; FT: fructose training; FA: fructose arginine. Data are reported as means±SE (one-way ANOVA).

All rats gained weight similarly throughout the experimental period (P=0.96) (Figure 2). Rats in the F group had more abdominal fat than those in the other groups (P=0.0201), and lean mass (%) was higher in the FT group compared to C, F, and FA groups (P<0.0001) (Table 1).

**Table 1:** Experimental model characterization.

	C		F		FT		FA		P
	Mean	SE	Mean	SE	Mean	SE	Mean	SE	
Abdominal fat (mg)	25.40	2.19	35.29^*#§^	3.18	24.96	1.95	25.97	2.02	0.0201
Lean mass (%)	10.71	0.76	10.01	0.56	18.50^*†§^	1.29	8.04	1.37	<0.0001
Glucose (mg/dL)	82.20	1.16	87.40	4.20	87.80	3.97	71.00^*†#^	2.26	0.0055
Insulin (mmol/dL)	1.10	0.13	1.93^*#§^	0.11	1.19	0.17	1.22	0.12	0.0093
Total cholesterol (mmol/L)	50.36	4.54	60.35	10.08	60.28	6.52	53.54	7.62	0.6833
HDL-c (mmol/L)	28.13	2.56	37.50	5.61	60.25^*†§^	11.43	34.50	4.98	0.0103
LDL-c (mmol/L)	22.11	4.02	26.25	9.45	28.42	11.96	19.66	3.46	0.8370
TAG (mmol/L)	81.18	9.51	302.8^*#§^	89.12	100.20	12.70	119.80	11.04	0.0117

Groups: C: control; F: fructose; FT: fructose training; FA: fructose arginine. HDL-C: high density lipoprotein cholesterol; LDL-C: low density lipoprotein cholesterol; TAG: triacylglycerol. *P<0.05 *vs* C; ^†^P<0.05 *vs* F; ^#^P<0.05 *vs* FT; ^§^P<0.05 *vs* FA (one-way ANOVA).

Regarding biochemical data, at the end of the experimental period, the FA group presented lower fasting glucose levels than the other groups (P=0.0055), whereas insulin levels were higher in the F group than in the other groups (P=0.0093). Total cholesterol and LDL-c levels were similar among groups (P=0.63833 and P=0.8370, respectively); however, HDL-c levels were higher in the FT group (P=0.0103). Moreover, the fructose-rich diet without any treatment induced an increase in triacylglycerol levels (P=0.0117 *vs* C, FT, and FA) (Table 1).

### MET parameters

Aerobic training was effective in improving the performance of rats in the MET (Table 2). Changes (Δ, final MET - initial MET) in distance, time, and maximum speed achieved were greater in the FT group than in the non-training groups (P=0.0075, P=0.0006, and P=0.0026, respectively).

**Table 2:** Maximal exercise test (MET) data.

	C		F		FT		FA		P
	Mean	SE	Mean	SE	Mean	SE	Mean	SE	
Δ Time	20.68	1.92	18.77	3.54	34.08^*†^	2.41	13.46^§^	4.27	0.0006
Δ Distance	0.43	0.06	0.39	0.09	0.82^*†^	0.14	0.32^§^	0.08	0.0075
Δ Maximum speed	1.63	0.05	1.60	0.18	2.34^*†^	0.11	1.33^§^	0.32	0.0026

Groups: C: control; F: fructose; FT: fructose training; FA: fructose arginine. n=7 animals per group. Δ: Difference between initial and final METs. *P<0.05 *vs* C; ^†^P<0.05 *vs* F; ^§^P<0.05 *vs* FT (one-way ANOVA).

### Protein expression

**Figure 3:**
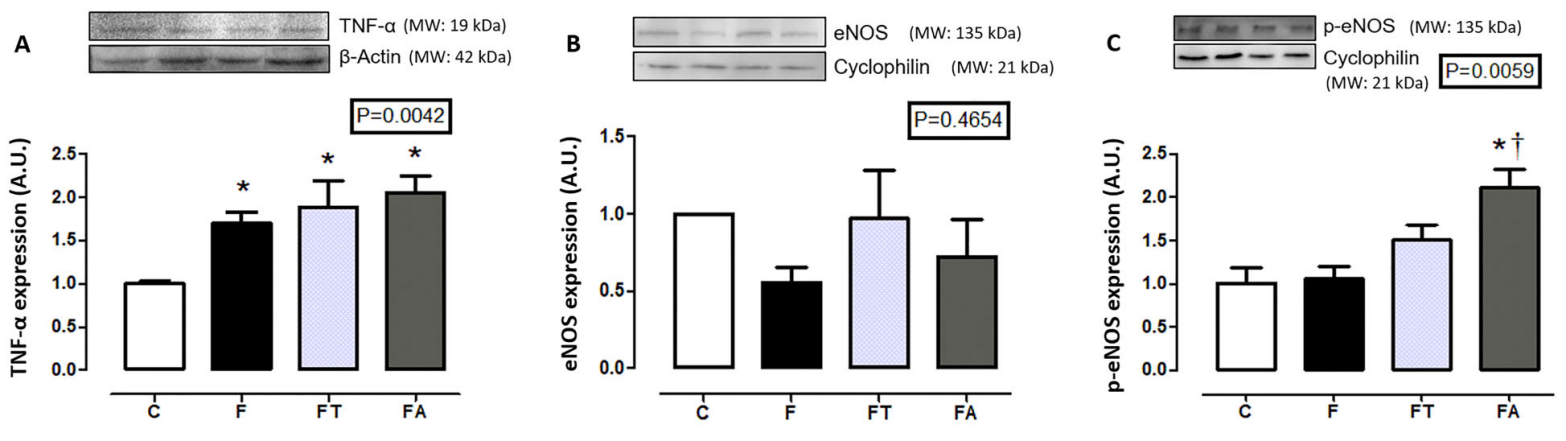
Protein expression of (**A**) tumor necrosis factor-alpha (TNF-α), (**B**) endothelial nitric oxide synthase (eNOS), and (**C**) phosphorylated endothelial nitric oxide synthase (p-eNOS) in the left ventricle tissue (n=7 per group). A.U.: arbitrary unit; MW: molecular weight. Groups: C: control; F: fructose; FT: fructose training; FA: fructose arginine. Data are reported as means±SE. *P<0.05 *vs* C; ^†^P<0.05 *vs* F (one-way ANOVA).

TNF-α expression was higher in the groups that received a fructose-rich diet (F, FT, and FA) than in the control group (P=0.0042). No alterations were observed in eNOS expression among groups (P=0.4654), whereas p-eNOS expression was higher in the FA group than in the C and F groups (P=0.0059) (Figure 3).

### Nitrite level, lipid peroxidation, and cardiac remodeling

No differences were observed in nitrite levels among the experimental groups (P=0.6024). Lipid peroxidation, verified through malondialdehyde assay, was greater in the F group than in the C and FT groups (P=0.0135). Cardiac remodeling, evaluated by MMP activation (pro-MMP-2 and MMP-2), was similar among the groups (P=0.7840 and P=0.1312, respectively) (Table 3).

**Table 3:** Nitrite, lipid peroxidation, and metalloproteinases results.

	C		F		FT		FA		P
	Mean	SE	Mean	SE	Mean	SE	Mean	SE	
Nitrite (nM)	365.40	64.00	352.10	63.00	425.30	101.10	540.60	180.10	0.6024
Malondialdehyde (nmol/mL)	15.78	1.60	21.77^*^	1.02	15.70^†^	1.27	17.82	1.63	0.0135
Pro-MMP-2 (A.U.)	0.96	0.07	1.03	0.13	0.94	0.13	0.87	0.12	0.7840
MMP-2 (A.U)	1.09	0.15	0.74	0.07	0.74	0.16	0.61	0.07	0.1213

All analyzes were performed in serum. Groups: C: control; F: fructose; FT: fructose training; FA: fructose arginine. n=7 animals per group. Pro-MMP-2: pro-metalloproteinase-2; MMP-2: inactive metalloproteinase-2. *P<0.0. *vs* C; ^†^P<0.05 *vs* F (one-way ANOVA). A.U.: arbitrary units.

### Histological analysis

**Figure 4:**
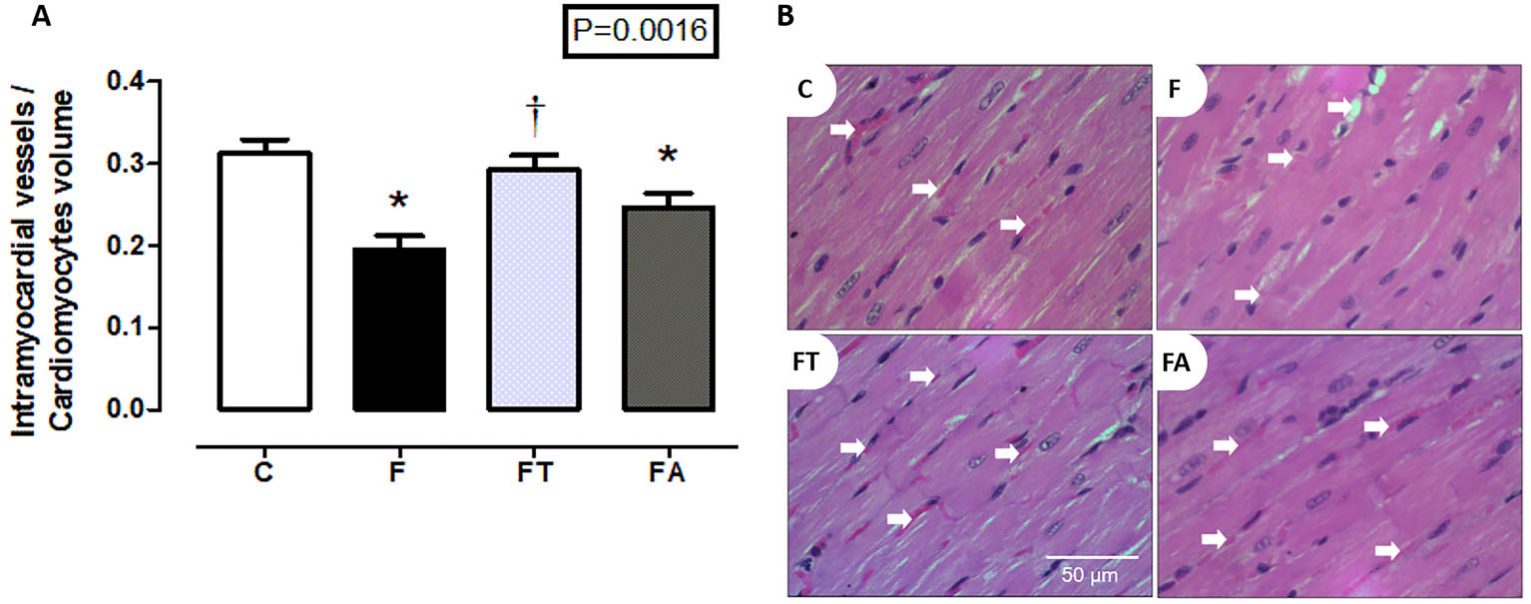
Left ventricle vascularization. **A**, Intramyocardial vessel/cardiomyocyte volume ratio (n=7 per group) of the control (C), fructose (F), fructose training (FT), and fructose arginine (FA) groups. **B**, Representative images of HE-stained longitudinal sections of intramyocardial vessels (arrows) of the groups. Scale bar 50 μm. Data are reported as means±SE. *P<0.05 *vs* C; ^†^P<0.05 *vs* F (one-way ANOVA).

Figure 4 shows the intramyocardial vessel/cardiomyocyte volume ratio, which indicates tissue vascularization. Myocardial vascularization was lower in the F and FA groups than in the C group, whereas it was higher in the FT group than in the F group (P=0.0016).

**Figure 5:**
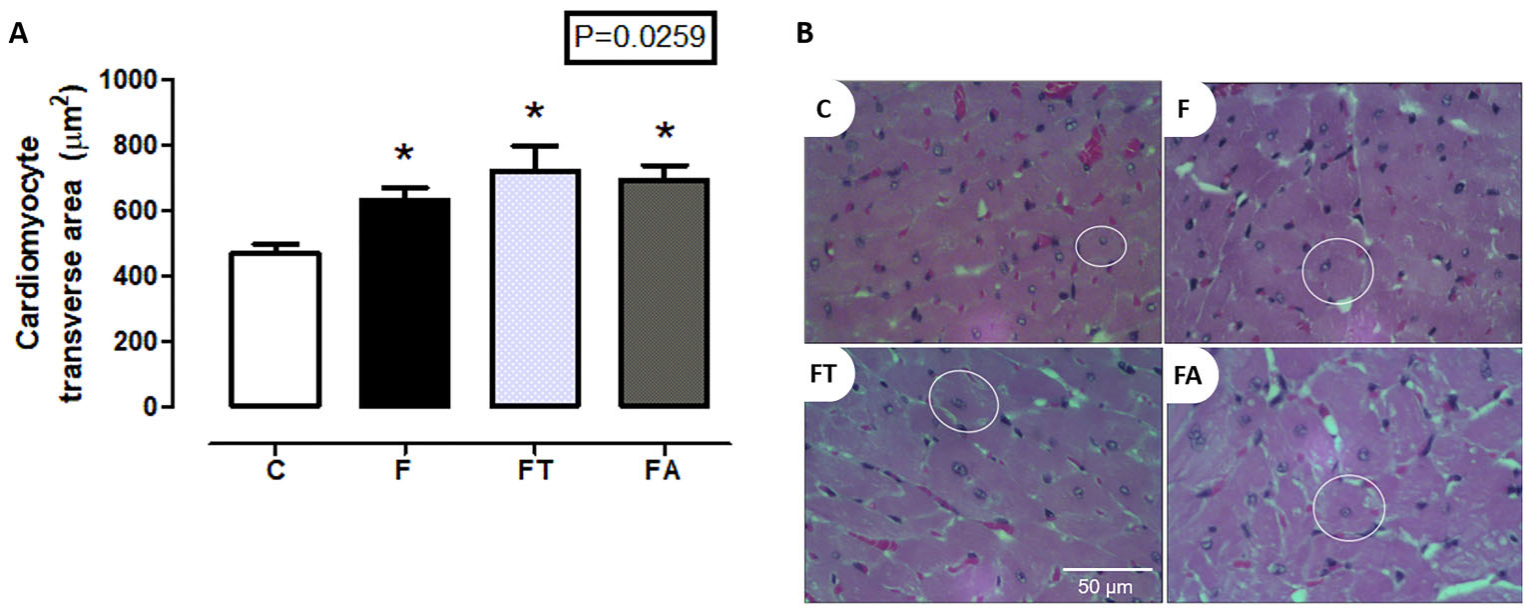
Cardiomyocyte area in the longitudinal section. **A**, Cardiomyocyte transverse area (µm^2^) (n=7 per group) ) of the control (C), fructose (F), fructose training (FT), and fructose arginine (FA) groups. B, Representative images of cardiomyocyte area in HE-stained transverse section of the groups. Circles indicate cardiomyocytes. Scale bar 50 μm. Data are reported as means±SE. *P<0.05 *vs* C (one-way ANOVA).

Figure 5 shows the longitudinal cardiomyocyte area. All fructose-fed groups (F, FT, and FA) showed cardiomyocyte hypertrophy compared with the C group (P=0.0259).

### MicroRNA quantification

MicroRNA quantification after 10 weeks is shown in Table 4. No significant differences were observed in the quantification of microRNA-126, -195, and -146a when groups were compared to C (P=0.1074, P=0.8308, and P=0.2361, respectively).

**Table 4:** MicroRNAs-126, -195, and -146a quantifications.

	C		F		FT		FA		P
	Mean	SE	Mean	SE	Mean	SE	Mean	SE	
MicroRNA-126 (% relative to C)	100.00	15.67	95.75	8.90	89.61	9.99	129.2	11.00	0.1074
MicroRNA-195 (% relative to C)	99.97	24.63	106.90	12.25	107.2	4.68	93.60	4.08	0.8308
MicroRNA-146a (% relative to C)	100.00	11.84	79.57	15.29	158.7	33.87	143.2	48.94	0.2361

All analyses were performed on the left ventricle (n=7 animals per group). Groups: C: control; F: fructose; FT: fructose training; FA: fructose arginine. No significant differences were observed among the groups (one-way ANOVA).

## Discussion

In the present study, we investigated for the first time the possible effects of non-pharmacological prevention strategies, such as L-arginine supplementation and aerobic physical training, for reversing the systemic pro-oxidative environment during the onset of CVD, avoiding metabolic and molecular damage in cardiomyocytes.

According to the literature (17), the high-fructose intake protocol led to a model of early cardiometabolic diseases, shown by an increase in systemic pro-oxidative status, abdominal fat mass, triacylglycerol level, and insulin level in rats. Additionally, in this experimental model, an increase in systemic oxidative stress, alterations in cardiomyocytes, such as lower LV vascularization, cardiac hypertrophy, and higher TNF-α expression, were observed.

Therefore, this study is a pioneer in trying to understand molecularly the impact of these damages in the myocardium and how non-pharmacological treatments could help prevention. Darband et al. (28) verified the attenuation of oxidative stress in the LV of aged Wistar rats after 12 weeks of exercise training and L-arginine supplementation. In this context, some beneficial alterations caused by moderate training and L-arginine supplementation were observed.

The aerobic training protocol was effective, since an improvement in the parameters of time, distance, and speed was observed only in the animals that were submitted to the training protocol. The trained group had improvement in HDL-c level, lean body mass, and lipid peroxidation, normalized insulin levels and abdominal fat mass, but there was no reduction in TNF-α levels.

With regard to histological analysis, training alone prevented fructose-induced LV vascularization reduction and no treatment prevented LV hypertrophy observed in all groups that received fructose. Similar to the previous result, pro-MMP-2 and MMP-2 activities did not differ among groups. These results are similar to those reported by Ranjbar et al. (29), who induced myocardial infarction in Wistar rats and treated them with treadmill running at moderate intensity, 50 min per session, 5 times a week, for 10 weeks. Arteriole density in the myocardium was higher in the infarcted groups that underwent physical training than in the controls.

It is also important to consider that constant fructose consumption leads to pathological cardiac hypertrophy, characterized by an abnormal thickening of the cardiac muscle, owing to pressure or volume overload triggered by conditions such as arterial hypertension, aortic stenosis, valvulopathies, and acute myocardial infarction (30). However, physical training promoted histological changes in the LV. Indeed, this type of change is considered a normal adaptive mechanism of the cardiac muscle in response to pressure or volume overload (31).

Even though L-arginine did not change the aerobic capacity of the animals, it normalized serum insulin, triacylglycerol, and circulating oxidative stress levels and abdominal fat content.

In the present study, although there were no changes in the circulating nitrite concentration with L-arginine supplementation, the expression of the active form of eNOS was increased in fructose-fed rats, but it was not enough to reverse the reduced vascularization caused by excess fructose. L-arginine is the main precursor of NO, a potent endothelium-derived vasodilator that plays an important role in controlling vascular tone in animals and humans (32). In this sense, the increase in vessel caliber may guarantee a greater blood flow and thus new vessel production would be secondary.

Increased TNF-α levels were observed in all groups that received fructose, and no treatment was able to reduce them. However, the present data demonstrated an increase in oxidative stress in animals with excess fructose in their diet. These results corroborate previous data (33) that high fructose intake leads to increased PKC activation, which leads to increased ROS production through PKC-dependent NADPH activation, culminating in superoxide anion free radical production, which has deleterious effects on proteins, lipids, and DNA. High exposure to these molecules promotes severe tissue damage, leading to cell death (34), which may explain the metabolic damage observed in animals fed excess fructose.

As mentioned, both aerobic training and L-arginine supplementation reduced systemic oxidative stress, which may explain the reduction in myocardial damage. A similar response was observed in men after 8 weeks of medium-to-high intensity aerobic training, who had decreased plasma concentrations of oxidative markers (35). L-arginine also reduced oxidative stress in animal studies. Ranjbar et al. (29) observed a reduction in malondialdehyde concentrations in the liver tissue of Wistar rats with myocardial infarction treated with L-arginine (4% in drinking water) or aerobic exercise for 10 weeks.

In the present study, in parallel to the maintenance of MMP-2 activity, there was a significant increase in the cardiomyocyte area in all fructose groups, indicating that less cardiac remodeling promotes preexisting tissue enlargement. The moment in the experimental timeline when the analyses were performed can also explain these results. Bellafiore et al. (36) reported that cardiomyocyte MMP-2 activity is reduced in healthy mice after 30 and 45 days of low-to-moderate intensity aerobic training. However, MMP-9 activity increased after 15 days of training and, after 30 and 45 days, it returned to baseline levels. It should be noted that in the present study, the evaluation of serum MMP-2 activity was verified after 60 days of training and L-arginine supplementation.

Regarding genetic factors underlying the observed cardiovascular alterations, although microRNA-126 activity leads to cardiac impairments, such as tissue ischemia and heart failure (37), it seems that its expression is more important in endothelial and hematopoietic progenitor cells (7), which may explain the absence of significant differences in cardiomyocytes in this experimental model. Khakdan et al. (38) evaluated the effect of a high-fructose and fat-rich diet and high-intensity interval training (HIIT) and reported that continuous endurance training influences microRNA-195 expression. A diet with 30% energetic value from lipids and 20% from fructose resulted in an increase in microRNA-195 expression compared with the control group, and physical training reduced its expression, with a greater magnitude of change in the HIIT group. However, the differences between protocols may explain the absence of significant differences in the present study.

Additionally, experimental studies have demonstrated that rodents with diabetes have reduced levels of microRNA-146a, and its dysregulation seems to occur with stimuli such as increased NF-κB, TNF-α, and IL-1β expression (39). However, the animals in our cardiometabolic disease onset model did not have diabetes, which may explain why higher TNF-α levels did not modify gene expression.

The results of this study must be interpreted in light of some limitations. It was not possible to confirm the differences among LV hypertrophies obtained in the fructose-fed groups; thus, other studies could contribute to the analysis of proteins, such as mTOR and protein kinase S6 (40). However, the myocardial vascularization does help in understanding the hypertrophy of the presented experimental model as a pathological process, and may clarify the understanding of how such non-pharmacological treatments can reverse or attenuate the pathological changes caused in the initial phase of CVD development.

In conclusion, excess consumption of fructose led to early stage CVD with cardiomyocyte damage, and moderate aerobic exercise improved the metabolic profile, decreased oxidative stress, and prevented the reduction of cardiomyocyte vascularity. L-arginine supplementation prevented metabolic changes, decreased oxidative stress, and promoted the activation of eNOS in cardiomyocytes. Thus, both treatments minimized the cardiometabolic impact of excess fructose during CVD progression, and cardiomyocyte alterations were independent of MMP-2 activity and microRNA-126, -195, and -146a expression and influenced by pro-oxidative environments.

## References

[1] Delbridge LM, Benson VL, Ritchie RH, Mellor KM (2016). Diabetic cardiomyopathy: the case for a role of fructose in disease etiology. Diabetes.

[2] Basciano H, Federico L, Adeli K (2005). Fructose, insulin resistance, and metabolic dyslipidemia. Nutr Metab (Lond).

[3] Rönn M, Lind PM, Karlsson H, Cvek K, Berglund J, Malmberg F (2013). Quantification of total and visceral adipose tissue in fructose-fed rats using water-fat separated single echo MRI. Obesity (Silver Spring).

[4] Zhao C, Zhang Y, Liu H, Li P, Zhang H, Cheng G (2017). Fortunellin protects against high fructose-induced diabetic heart injury in mice by suppressing inflammation and oxidative stress via AMPK/Nrf-2 pathway regulation. Biochem Biophys Res Commun.

[5] Sawyer DB, Siwik DA, Xiao L, Pimentel DR, Singh K, Colucci WS (2002). Role of oxidative stress in myocardial hypertrophy and failure. J Mol Cell Cardiol.

[6] Mann DL (2015). Innate immunity and the failing heart: the cytokine hypothesis revisited. Circ Res.

[7] Nazari-Jahantigh M, Egea V, Schober A, Weber C (2015). MicroRNA-specific regulatory mechanisms in atherosclerosis. J Mol Cell Cardiol.

[8] Halliwill JR, Minson CT, Joyner MJ (2000). Effect of systemic nitric oxide synthase inhibition on postexercise hypotension in humans. J Appl Physiol (1985).

[9] Kahn SE, Hull RL, Utzschneider KM (2006). Mechanisms linking obesity to insulin resistance and type 2 diabetes. Nature.

[10] Teixeira-Lemos E, Nunes S, Teixeira F, Reis F (2011). Regular physical exercise training assists in preventing type 2 diabetes development: focus on its antioxidant and anti-inflammatory properties. Cardiovasc Diabetol.

[11] Álvares T, Meirelles C, Bhambhani Y, Paschoalin V, Gomes P (2011). L-Arginine as a potential ergogenic aid in healthy subjects. Sports Med.

[12] Adams MR, Jessup W, Celermajer DS (1997). Cigarette smoking is associated with increased human monocyte adhesion to endothelial cells: reversibility with oral L-arginine but not vitamin C. J Am Coll Cardiol.

[13] Miczke A, Suliburska J, Pupek-Musialik D, Ostrowska L, Jablecka A, Krejpcio Z (2015). Effect of L-arginine supplementation on insulin resistance and serum adiponectin concentration in rats with fat diet. Int J Clin Exp Med.

[14] Medeiros RF, Gaique TG, Bento-Bernardes T, Kindlovits R, Gomes TMB, Motta NAV (2017). Arginine and aerobic training prevent endothelial and metabolic alterations in rats at high risk for the development of the metabolic syndrome. Br J Nutr.

[15] Reagan-Shaw S, Nihal M, Ahmad N (2008). Dose translation from animal to human studies revisited. FASEB J.

[16] Siani A, Pagano E, Iacone R, Iacoviello L, Scopacasa F, Strazzullo P (2000). Blood pressure and metabolic changes during dietary L-arginine supplementation in humans. Am J Hypertens.

[17] Medeiros RF, Gaique TG, Bento-Bernardes T, Motta NA, Brito FCF, Fernandes-Santos C (2016). Aerobic training prevents oxidative profile and improves nitric oxide and vascular reactivity in rats with cardiometabolic alteration. J Appl Physiol (1985).

[18] Kulics JM, Collins HL, DiCarlo SE (1999). Postexercise hypotension is mediated by reductions in sympathetic nerve activity. Am J Physiol.

[19] de Oliveira MAB, Brandi AC, dos Santos CA, Botelho PHH, Cortez JLL, de Godoy MF (2014). Comparison of fractal dimension and Shannon entropy in myocytes from rats treated with histidine-tryptophan-glutamate and histidine-tryptophan cetoglutarate. Rev Bras Cir Cardiovasc.

[20] Kahn SE, Larson VG, Beard JC, Cain KC, Fellingham GW, Schwartz RS (1990). Effect of exercise on insulin action, glucose tolerance, and insulin secretion in aging. Am J Physiol.

[21] Friedewald WT, Levy RI, Fredrickson DS (1972). Estimation of the concentration of low-density lipoprotein cholesterol in plasma, without use of the preparative ultracentrifuge. Clin Chem.

[22] Gomes VA, Casella-Filho A, Chagas AC, Tanus-Santos JE (2008). Enhanced concentrations of relevant markers of nitric oxide formation after exercise training in patients with metabolic syndrome. Nitric Oxide.

[23] Janero DR (1990). Malondialdehyde and thiobarbituric acid-reactivity as diagnostic indices of lipid peroxidation and peroxidative tissue injury. Free Radic Biol Med.

[24] Tschanz SA, Burri PH, Weibel ER (2011). A simple tool for stereological assessment of digital images: the STEPanizer. J Microsc.

[25] Storch AS, Rocha HNM, Garcia VP, Batista GMS, Mattos JD, Campos MO (2018). Oscillatory shear stress induces hemostatic imbalance in healthy men. Thromb Res.

[26] Fernandes T, Magalhães FC, Roque FR, Phillips MI, Oliveira EM (2012). Exercise training prevents the microvascular rarefaction in hypertension balancing angiogenic and apoptotic factors: role of microRNAs-16, -21, and -126. Hypertension.

[27] Souza LL, Cordeiro A, Oliveira LS, de Paula GS, Faustino LC, Ortiga-Carvalho TM (2011). Thyroid hormone contributes to the hypolipidemic effect of polyunsaturated fatty acids from fish oil: *in vivo* evidence for cross talking mechanisms. J Endocrinol.

[28] Darband SG, Sadighparvar S, Yousefi B, Kaviani M, Mobaraki K, Majidinia M (2020). Combination of exercise training and L-arginine reverses aging process through suppression of oxidative stress, inflammation, and apoptosis in the rat heart. Pflugers Arch.

[29] Ranjbar K, Rahmani-Nia F, Shahabpour E (2016). Aerobic training and l-arginine supplementation promotes rat heart and hindleg muscles arteriogenesis after myocardial infarction. J Physiol Biochem.

[30] Fan XD, Wan LL, Duan M, Lu S (2018). HDAC11 deletion reduces fructose-induced cardiac dyslipidemia, apoptosis and inflammation by attenuating oxidative stress injury. Biochem Biophys Res Commun.

[31] Garcia JA, Incerpi EK (2008). Factors and mechanisms involved in left ventricular hypertrophy and the anti-hypertrophic role of nitric oxide. Arq Bras Cardiol.

[32] Kiowski W (1991). Endothelial function in humans. Studies of forearm resistance vessels. Hypertension.

[33] Busserolles J, Gueux E, Rock E, Demigné C, Mazur A, Rayssiguier Y (2003). Oligofructose protects against the hypertriglyceridemic and pro-oxidative effects of a high fructose diet in rats. J Nutr.

[34] Evans JL, Goldfine ID, Maddux BA, Grodsky GM (2002). Oxidative stress and stress-activated signaling pathways: a unifying hypothesis of type 2 diabetes. Endocr Rev.

[35] Santilli F, Vazzana N, Iodice P, Lattanzio S, Liani R, Bellomo RG (2013). Effects of high-amount-high-intensity exercise on *in vivo* platelet activation: modulation by lipid peroxidation and AGE/RAGE axis. Thrombosis and haemostasis.

[36] Bellafiore M, Battaglia G, Bianco A, Farina F, Palma A, Paoli A (2013). The involvement of MMP-2 and MMP-9 in heart exercise-related angiogenesis. J Transl Med.

[37] Condorelli G, Latronico MVG, Dorn GW (2010). microRNAs in heart disease: putative novel therapeutic targets?. Eur Heart J.

[38] Khakdan S, Delfan M, Heydarpour Meymeh M, Kazerouni F, Ghaedi H, Shanaki M (2020). High-intensity interval training (HIIT) effectively enhances heart function via miR-195 dependent cardiomyopathy reduction in high-fat high-fructose diet-induced diabetic rats. Arch Physiol Biochem.

[39] Feng B, Chen S, Gordon AD, Chakrabarti S (2017). miR-146a mediates inflammatory changes and fibrosis in the heart in diabetes. J Mol Cell Cardiol.

[40] Brooks GA, White TP (1978). Determination of metabolic and heart rate responses of rats to treadmill exercise. J Appl Physiol Respir Environ Exerc Physiol.

